# Impact of sex in axial spondyloarthritis: insights from the Brazilian Registry of Spondyloarthritis

**DOI:** 10.1186/s42358-026-00525-3

**Published:** 2026-02-12

**Authors:** C. D. L. Marques, G. G. Resende, M. M. Pinheiro, C. G. S. Saad, A. O. Marinho, A. M. Soares, B. E. Paiva, C. P. Albuquerque, D. L. N. Rodrigues, G. R. W. Castro, G. A. Bulbol, J. N. Carneiro, J. M. C. Fernandes, M. L. G. Ochtrop, M. B. R. O. Gavi, M. E. G. Veiga, M. A. Yazbek, N. G. Cavalcanti, N. P. Machado, O. B. Malheiro, R. B. Macedo, R. M. R. A. Vieira, R. C. Lage, R. C. Menin, R. T. M. Golebiovski, S. L. E. Ribeiro, T. L. Oliveira, V. G. Dinis, P. D. Sampaio-Barros

**Affiliations:** 1 Hospital das Clínicas da Universidade Federal de Pernambuco (UFPE), Recife, Brazil; 2 Hospital das Clínicas da Universidade Federal de Minas Gerais (UFMG), Belo Horizonte, Brazil; 3 Escola Paulista de Medicina (EPM) - Universidade Federal de São Paulo (UNIFESP), São Paulo, Brazil; 4 Faculdade de Medicina da Universidade de São Paulo (USP), São Paulo, Brazil; 5 Fundação Hospital do Acre, Rio Branco, Brazil; 6 Hospital Universitário da Universidade Federal de Santa Catarina (UFSC), Florianópolis, Brazil; 7 Faculdade de Medicina da Universidade Federal do Amazonas (UFAM), Manaus, Brazil; 8 Hospital Universitário de Brasília, PPG em Ciências Médicas, FM/UnB, Brasíli, Brazil; 9 Hospital Governador Celso Ramos (HCR), Florianópolis, Brazil; 10 Hospital de Base do Distrito Federal (HBDF), Brasília, Brazil; 11 Universidade Federal do Maranhão (UFMA), São Luis, Brazil; 12 Hospital Universitário Pedro Ernesto / UERJ, Rio de Janeiro, Brazil; 13 Universidade Federal do Espírito Santo (UFES), Vitória, Brazil; 14 Universidade Estadual de Campinas (UNICAMP), São Paulo, Brazil; 15 Universidade Federal do Paraná (UFPR), Curitiba, Brazil; 16 Universidade Estadual do Ceará (UECE), Fortaleza, Brazil; 17 Faculdade de Medicina de São José do Rio Preto (FAMERP), São Paulo, Brazil

**Keywords:** Axial spondyloarthritis, Sex differences, Disease burden

## Abstract

**Background:**

Sex differences in axial spondyloarthritis (axSpA) are increasingly recognized, with women often reporting higher disease burden despite similar objective inflammatory markers. This study aimed to compare clinical features, disease activity, function, quality of life, and treatment patterns between men and women with axSpA and identify sex-specific predictors of disease outcomes using data from the Brazilian Registry of Spondyloarthritis (RBE).

**Methods:**

This was a cross-sectional, observational study based on data from the RBE, a nationwide multicenter cohort including 828 patients (568 men and 260 women) from 17 referral centers across Brazil. Standardized clinical and demographic data were collected using the REDCap platform. Disease activity (ASDAS-CRP, BASDAI), physical function (BASFI), spinal mobility (BASMI), and quality of life (ASQoL) were assessed with validated instruments. Sex-stratified multivariable linear regression models were constructed to identify independent predictors of each outcome.

**Results:**

Women presented higher disease activity (median BASDAI 4.5 vs. 3.2; ASDAS-CRP 2.2 vs. 1.9), greater functional limitation (BASFI 5.0 vs. 4.0), and poorer quality of life (ASQoL 9.0 vs. 7.0) compared to men, despite similar CRP levels. Psychological distress was more frequent in women, while men had worse spinal mobility (BASMI 4.0 vs. 3.5) and higher HLA-B27 positivity. Regression models revealed that shoulder and hip pain were relevant predictors of disease activity in both sexes, but psychological factors and work activity more strongly influenced outcomes in women. Men’s disease burden was more associated with structural damage and cardiometabolic comorbidities.

**Conclusions:**

This study highlights distinct sex-related clinical patterns in axSpA. Women reported higher symptom burden, functional limitations, and reduced quality of life, largely influenced by subjective symptoms and comorbidities. Conversely, men presented greater structural impairment and different comorbidity profiles. These findings support the need for sex-informed clinical assessments and individualized management strategies in axSpA to tailor the care of these sex-specific disease trajectories, which may enhance equity and outcomes in real-world settings.

**Supplementary Information:**

The online version contains supplementary material available at 10.1186/s42358-026-00525-3.

## Introduction

Axial Spondyloarthritis (axSpA) has historically been recognized to affect men more frequently and severely than women, with early studies estimating a tenfold higher prevalence of axial involvement in men vs. women [1, 2]. This sex disparity has decreased over time as the disease manifestations became better understood and diagnostic tools better interpreted. Recent data indicates a prevalence male: female ratio ranging from 1.2 to 2:1 [3]. This growing recognition of sex-based variation in axSpA, increasingly suggesting that the burden of axSpA in women may be underestimated, has prompted important clinical questions.

Women with axSpA often face a more complex journey to diagnosis. They are more frequently misdiagnosed, experience longer delays before receiving a correct diagnosis and tend to present with different initial symptoms compared to men [3, 4]. Their disease course may also differ, with unique challenges related to symptom burden, treatment response, and adherence, including leading women to be more likely to discontinue biologic therapies earlier than men [3, 4]. Despite these differences, sex-specific data on axSpA remain scarce, leaving many questions unanswered about how the disease truly manifests and progresses in women.

The need to better understand sex-based differences in axSpA has become especially urgent in the context of personalized medicine and equity-driven care. As the field moves toward more individualized treatment strategies, acknowledging and addressing these differences is essential to ensure that both men and women receive accurate diagnoses, timely interventions, and optimal disease management [5].

In this context, we must clearly differentiate the terms ‘sex’ and ‘gender.’ Although often used interchangeably, they represent distinct concepts: ‘sex’ refers to biological and physiological differences, whereas ‘gender’ encompasses social and cultural roles, expectations, and behaviors. This distinction is key to accurately interpreting disease patterns and outcomes [3]. For the purpose of this study, we refer specifically to ‘sex’ when comparing male and female patients with axSpA.

This study aims to investigate sex-based differences in the clinical presentation and disease burden in patients with axSpA. Drawing on data from the Brazilian Registry of Spondyloarthritis (RBE), the largest nationally representative cohort in Latin America, we aim to generate robust, sex-stratified evidence on axSpA to uncover clinically relevant differences that may support more accurate diagnoses, individualized treatment strategies, and equitable care for both men and women.

## Methods

The RBE is a multicenter, observational, and prospective cohort that has enrolled patients with SpA from 17 centers representing all five geographic regions of Brazil, as described elsewhere [6]. Briefly, patients treated at referral centers could be included if they met the following inclusion criteria: (1) had a diagnosis of SpA by an expert rheumatologist (axSpA, psoriatic arthritis, enteropathic arthritis, reactive arthritis or undifferentiated SpA); (2) age greater than or equal to 18 years; and (3) were classified according to the ASAS axial or peripheral criteria [7, 8]. Data were collected using a standardized protocol on the REDCap platform and included demographic information, HLA-B27 status, and clinical disease parameters, including peripheral and axial involvement, patient and physician-reported outcomes, extra-musculoskeletal manifestations, comorbidities, and treatment history. Psychological distress was defined as the presence of depressive and/or anxiety symptoms, based on self-reported medical history, clinical diagnosis, or current use of antidepressant or anxiolytic medication prescribed by a physician. Disease activity was assessed using the Bath Ankylosing Spondylitis Disease Activity Index (BASDAI) [9] and the Ankylosing Spondylitis Disease Activity Score using C-reactive protein (ASDAS-CRP) [10]. Other validated instruments, such as Bath Ankylosing Spondylitis Functional Index (BASFI) [11], Bath Ankylosing Spondylitis Metrology Index (BASMI) [12], Ankylosing Spondylitis Quality of Life (ASQoL) [13, 14] and modified Stoke Ankylosing Spondylitis Spinal Score (mSASSS) [14] and ASAS NSAID intake score [15] were also measured. Tender and swollen joint counts, as well as enthesitis sites, were also assessed through a standardized physical examination. For the present analysis, only patients classified as having axSpA were included.

### Statistical analysis

Descriptive statistics were computed for all variables. Continuous variables were summarized as medians and interquartile ranges, and categorical variables as frequencies and percentages. Comparisons between groups were performed using appropriate statistical tests according to data distribution and variable type. A p-value < 0.05 was considered statistically significant. Sex-stratified multivariable linear regression models were constructed to identify independent predictors of disease activity (ASDAS-CRP and BASDAI), spinal mobility (BASMI), physical function (BASFI), and quality of life (ASQoL). A multivariable binomial logistic regression model was used to identify factors independently associated with sex. In the initial model, male sex was set as the reference category. Consequently, odds ratios (ORs) below 1 reflected a lower likelihood among men and, indirectly, a higher likelihood among women. To facilitate interpretation and align the results with the study’s focus on sex-related differences—particularly the burden observed in women—we inverted the ORs (1/OR). This transformation does not affect the underlying model, statistical significance, or confidence intervals, but allows results to be presented with female sex as the reference category, such that OR > 1 indicates an association with female sex.

Variables included in each model were selected based on clinical relevance and bivariate associations with *p* < 0.20. Model performance was assessed using R, R², and adjusted R². Results were reported as unstandardized beta coefficients with 95% confidence intervals. Missing data were assessed for all variables. Those included in the multivariable models had < 10% missingness, and no systematic patterns were identified. Therefore, we conducted complete case analyses without imputation. Statistical analyses were performed using SPSS (version 29.0.2.0) and Jamovi (version 2.6.26), and graphical outputs were generated with GraphPad Prism (version 10). All details of the statistical analysis are provided in the supplementary material.

## Results

A total of 828 axSpA patients were included in the analysis, comprising 568 (68.6%) males and 260 (31.4%) females. Table 1 summarizes the demographic and clinical data of the sample, comparing men and women. Regarding demographic data, women had a later median age at symptom onset (29 years, IQR 19–34 vs. 27 years, IQR 21–38; *p* = 0.001) and age at diagnosis (37 years, IQR 30–47 vs. 35 years, IQR 27–44; *p* = 0.002), but shorter disease duration (16 years, IQR 9–26 vs. 18 years, IQR 10–28; *p* = 0.041) when compared to men. Despite these differences, diagnostic delay was similar between sexes (median 5 years, IQR 1–13 for both; *p* = 0.975). Although HLA-B27 positivity was more frequent among males (77.6% vs. 69.2%; *p* = 0.009; OR = 1.33, 95%CI 1.07–1.64), family history of SpA was more frequently reported by females (26.2% vs. 17.2%; *p* = 0.002; OR = 1.71, 95%CI 1.21–2.40).

While axial symptoms presented similar frequencies in both sexes, active peripheral involvement was more common in females (25.3% vs. 19.1%; *p* = 0.040; OR = 1.43, 95%CI 1.01–2.02). Emphasizing this finding, upper limb arthritis was significantly more reported by females (21.6% vs. 14.8%; *p* = 0.010; OR = 1.58, 95% CI 1.11–2.25). Concerning extra-musculoskeletal manifestations, no statistically significant differences were found between the sexes. Regarding comorbidities, psychological distress was more prevalent among females (43.8% vs. 20.9%; *p* < 0.001; OR = 2.95 95%CI 2.16–4.01) while chronic kidney disease was more frequently reported by males (3.0% vs. 0.7%; *p* = 0.028; OR = 0.22, 95%CI 0.05–0.96) (Table 1).

The prevalence of arterial hypertension, diabetes and obesity was similar between sexes. Psychological distress was more prevalent among females (43.8% vs. 20.9%; *p* < 0.001; OR = 2.95 95%CI 2.16–4.01) and chronic kidney disease was more frequently reported among males (3.0% vs. 0.7%; *p* = 0.028; OR = 0.22, 95%CI 0.05–0.96).

The overall proportion of physically active individuals did not differ significantly between sexes. However, a greater proportion of men reported engaging in physical exercise with higher frequency, specifically 4–7 times per week (45.7% vs. 29.9%). Regarding employment status, men were significantly more actively employed (48.9% vs. 32.3%; *p* < 0.001; OR = 0.27, 95% CI 0.17–0.41), as well as were more frequently retired due to disability (63.6% vs. 28.5%; *p* < 0.001; OR = 2.06, 95% CI 1.63–2.60) among those who were not currently working (Table 1).

There were no significant differences in prior treatment patterns. Regarding current treatments, sulfasalazine was more commonly used by men (16.0% vs. 10.7%; *p* = 0.029 ; OR = 0.62, 95%CI 0.40–0.95), and oral corticosteroid use was more frequent among women (4.5% vs. 2.1%; *p* = 0.042; OR = 2.18, 95%CI 1.01–4.70). Table 1 summarizes the demographic and clinical data of the sample, comparing men and women (Table 1).

Sex-stratified analysis of continuous clinical variables is presented in Table 2. Women had a later median age at symptom onset (29 years, IQR 21–38) compared to men (27 years, IQR 19–34) (*p* = 0.001) and were also diagnosed at an older age (37 years, IQR 30–47 vs. 35 years, IQR 27–44; *p* = 0.002). Despite these differences, diagnostic delay was similar between sexes (median 5 years, IQR 1–13 for both; *p* = 0.975). There were no significant differences in CRP levels, enthesitis count, mSASSS, ASAS NSAID intake score, swollen joint count (SJC), or tender joint count (TJC). However, VAS PGA (5.0, IQR 3.5–7.0 vs. 5.0, IQR 2.0–7.0; *p* < 0.001), pain (VAS 5.0, IQR 3.0–7.0 vs. 4.0, IQR 2.0–7.0; *p* < 0.001), and VAS MGA (4.0, IQR 2.0–6.0 vs. 3.0, IQR 1.0–5.0; *p* = 0.006) were all higher in women.

**Fig. 1:**
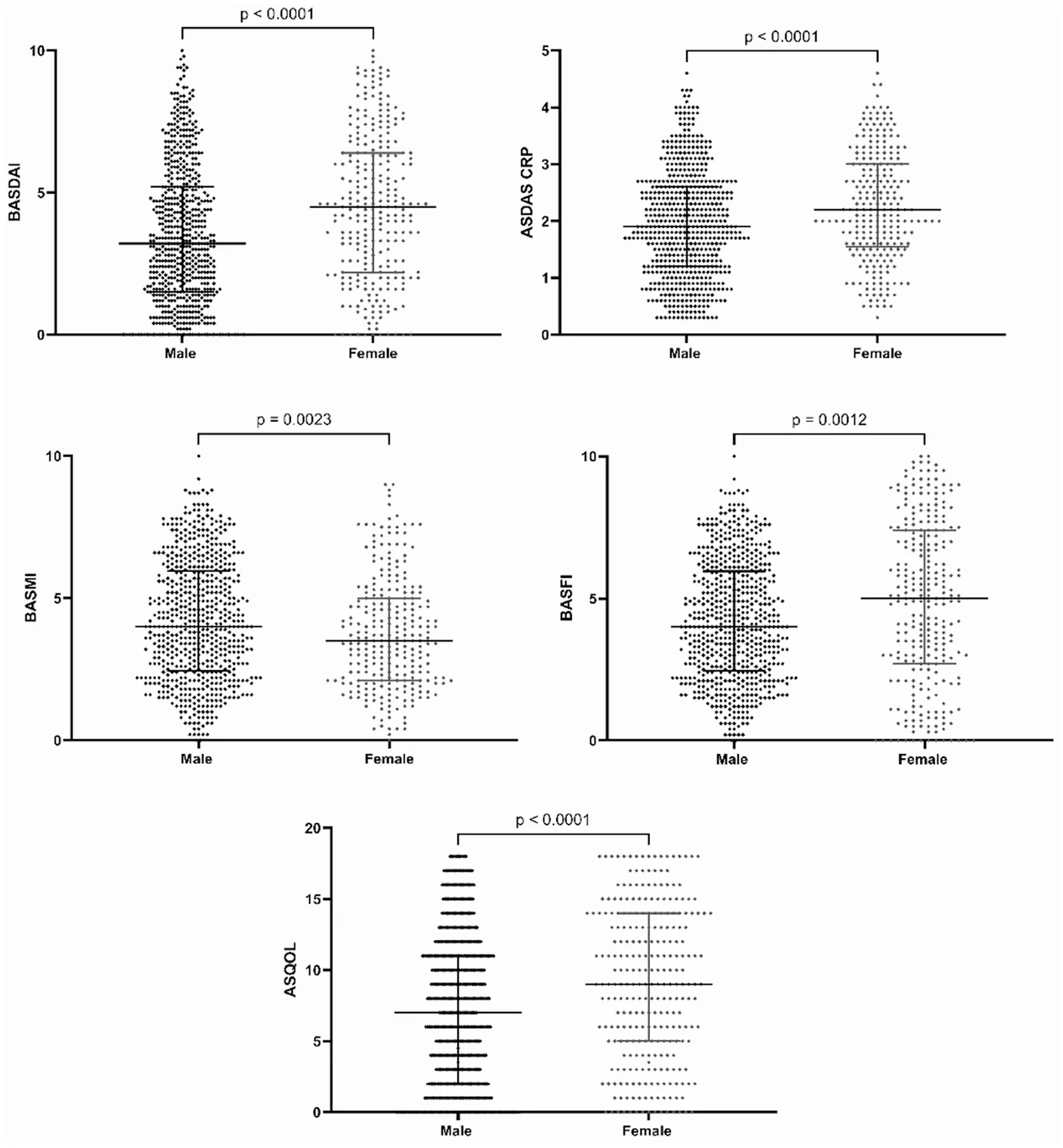
Sex-based comparison of disease activity, function, mobility, and quality of life in patients with axSpA. Dot plots illustrating sex differences in disease activity (BASDAI and ASDAS-CRP), functional status (BASFI), spinal mobility (BASMI), and quality of life (ASQoL) among patients with axSpA. Horizontal lines represent median and interquartile ranges ; p-values reflect group comparisons using multiple comparisons post-hoc tests following ANOVA or Kruskal-Wallis analysis (95%CI). BASDAI: Bath Ankylosing Spondylitis Disease Activity Index. ASDAS-CRP: Ankylosing Spondylitis Disease Activity Score based on C-reactive protein. BASMI: Bath Ankylosing Spondylitis Metrology Index. BASFI: Bath Ankylosing Spondylitis Functional Index. ASQoL: Ankylosing Spondylitis Quality of Life questionnaire

Sex-related differences in key clinical outcomes are illustrated in Fig. 1. Female patients had significantly higher disease activity scores, both by BASDAI (median 4.5 vs. 3.2; *p* < 0.0001) and by ASDAS-CRP (median 2.2 vs. 1.9; *p* < 0.0001). Functional impairment, assessed by BASFI (median 5.0 vs. 4.0; *p* = 0.0012), and quality of life, measured by ASQoL (median 9.0 vs. 7.0; *p* < 0.0001), were also worse in women. In contrast, spinal mobility assessed by BASMI was more restricted in men (median 4.0 vs. 3.5; *p* = 0.0023).

Sex-stratified bivariate analyses showed that musculoskeletal manifestations, particularly shoulder and hip pain and respective functional limitations, reflecting root-joint involvement, were significantly associated with worse BASDAI, ASDAS-CRP, BASFI, BASMI, and ASQoL scores in both men and women. Among male patients, additional factors consistently associated with worse outcomes across multiple domains included physical inactivity, not currently working, active peripheral arthritis, and comorbidities such as hypertension, diabetes, smoking, and obesity. In female patients, psychological distress was significantly associated with higher BASFI and ASQoL scores, while obesity and reduced physical activity were also linked to worse ASQoL. Detailed results are presented in the supplementary material.

**Fig. 2:**
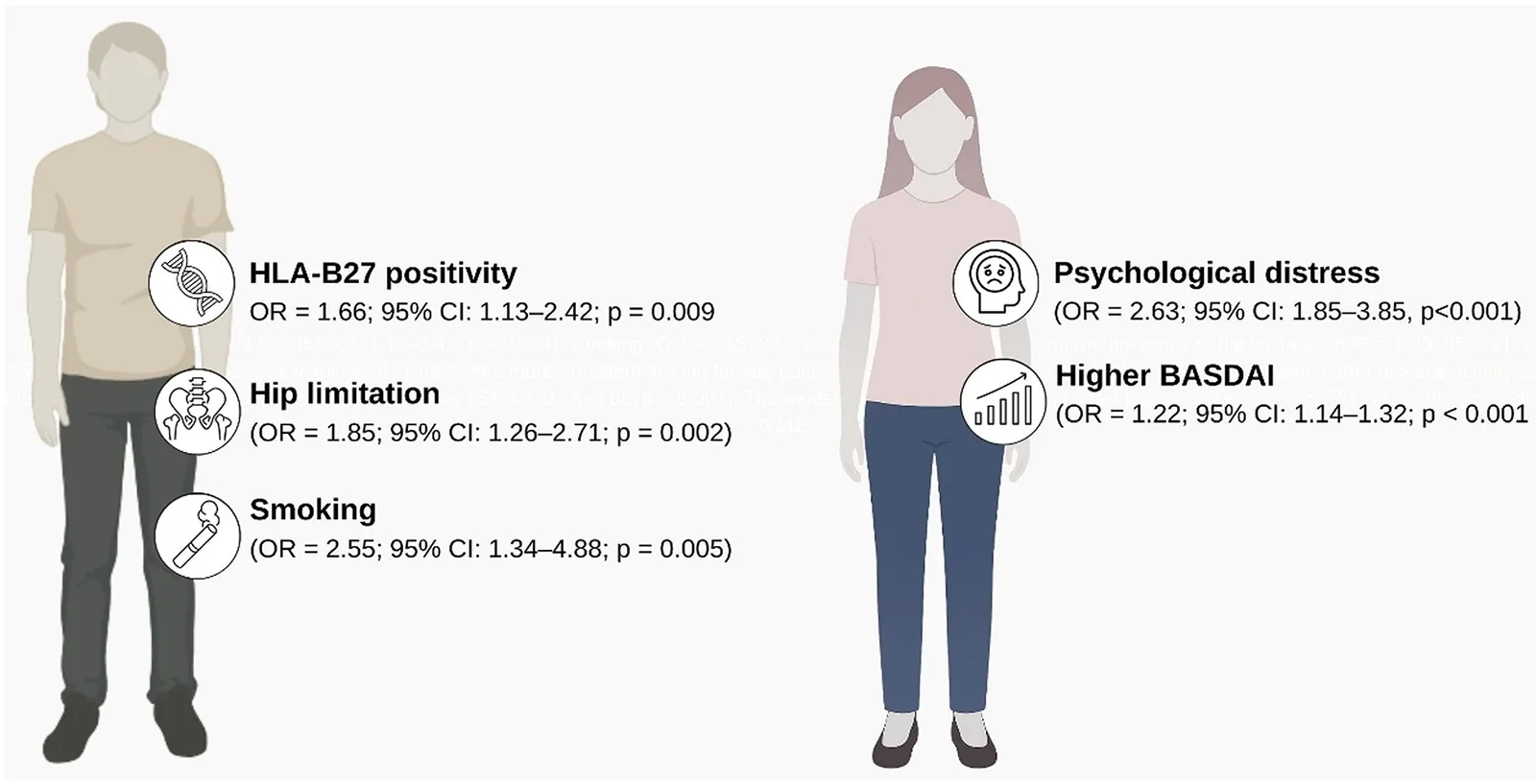
Independent variables associated with male and female sex in axial spondyloarthritis. Factors independently associated with male (left) and female (right) sex in patients with axial spondyloarthritis, based on multivariable binomial logistic regression analysis. Male sex was significantly associated with HLA-B27 positivity, hip limitation, and smoking, while female sex was associated with psychological distress and higher BASDAI scores. Model fit: deviance = 770; AIC = 782; pseudo R² (Nagelkerke) = 0.118. OR = odds ratio; CI = confidence interval

In the multivariable binomial logistic regression model, male sex was significantly associated with HLA-B27 positivity (OR = 1.66; 95%CI 1.13–2.42; *p* = 0.009), smoking (OR = 2.55; 95%CI 1.34–4.88; *p* = 0.005), and hip limitation (OR = 1.85; 95%CI 1.26–2.71; *p* = 0.002). In contrast, female patients was associated with psychological distress (OR = 2.63; 95% CI: 1.85–3.85; *p* < 0.001) and with higher disease activity scores as measured by BASDAI (OR = 1.22 per unit decrease; 95% CI: 1.14–1.32; *p* < 0.001) (Fig. 2).

**Table 1:** Comparative analysis between male (*n* = 568) and female (*n* = 260) patients with AxSpA across demographic, clinical and disease-related variables

	Male (*n* = 568)		Female (*n* = 260)		*p*	OR (95%CI)
	*n*	%	*n*	%		
**Skin color**						
*White*	279	41.9	121	41.6	0.928	0.98 (0.74–1.30)
*Non White*	387	58.1	170	58.4		
Active at work*	301	48.9	84	32.1	**0.001**	0.27 (0.17–0.41)
Retired due to illness**	196	63.6	74	28.5	**< 0.001**	2.06 (1.63–2.60)
**Comorbidities**						
*Hypertension*	238	35.7	103	35.4	0.932	1.00 (0.82–1.23)
*Diabetes*	95	14.2	37	12.7	0.528	0.87 (0.58–1.31)
*Obesity*	61	9.1	25	8.6	0.783	0.93 (0.57–1.52)
*Chronic renal diseases*	20	3.0	2	0.7	**0.028**	0.22 (0.05–0.96)
*Coronary heart disease*	9	1.3	5	1.7	0.662	1.27 (0.42–3.84)
*Psychological distress*	125	20.9	121	43.8	**< 0.001**	2.95 (2.16–4.01)
*Anxiety* ^*#*^	63	9.3	54	18.6	**< 0.001**	2.22 (1.50–3.30)
*Depression* ^*#*^	37	5.5	52	17.9	**< 0.001**	3.77 (2.41–5.89)
*No comorbidities*	299	44.8	102	35.1	**0.005**	0.66 (0.49–0.88)
**Smoking**	78	12.2	19	6.6	**0.010**	1.64 (1.08–2.49)
**Alcoholism**	41	6.5	4	1.4	**< 0.001**	3.63 (1.41–9.30)
**Physically active**	270	43.8	118	41.8	0.577	0.92 (0.69–1.22)
*1–3 times/week*	146	54.3	82	70.1	**0.004**	0.50 (0.31–0.80)
*4–7 times/week*	123	45.7	35	29.9		
**SpA family history**	106	17.2	73	26.2	**0.002**	1.71 (1.21–2.40)
**HLA-B27 positive**	441	77.6	180	69.2	**0.009**	1.33 (1.07–1.64)
**First symptoms**						
*Cervical pain*	236	35.4	92	31.6	0.258	1.12 (0.91–1.38)
*Hip pain*	151	22.6	62	21.3	0.648	0.92 (0.66–1.29)
*Dactylitis*	29	4.3	12	4.1	0.875	1.04 (0.64–1.69)
*Buttock pain*	257	38.5	120	41.2	0.430	1.12 (0.84–1.48)
*Enthesitis*	166	24.9	64	22.0	0.335	0.85 (0.61–1.18)
*Low back pain*	583	87.4	247	84.9	0.291	0.80 (0.54–1.19)
*Peripheral arthritis*	278	41.7	125	43.0	0.713	1.05 (
**EMM**						
*Uveitis*	203	30.4	98	33.7	0.320	1.16 (0.86–1.57)
*IBD*	16	2.4	12	4.1	0.145	1.75 (0.81–3.74)
*Psoriasis*	9	1.3	9	3.1	0.068	2.33 (0.91–5.9)
**Active peripheral involvement**	111	19.1	68	25.3	**0.040**	1.43 (1.01–2.02)
**Previous treatment**						
*NSAID*	287	43.0	123	42.3	0.827	0.96 (0.73–1.28)
*Methotrexate*	168	25.2	72	24.7	0.884	0.97 (0.71–1.34)
*TNFi*	236	35.4	115	39.5	0.222	1.19 (0.89–1.58)
*SSZ*	210	31.5	90	30.9	0.864	0.97 (0.72–1.31)
*Oral corticosteroid*	55	8.2	28	9;6	0.486	1.18 (0.73–1.91)
*IL17i*	15	2.2	10	3.4	0.289	1.54 (0.68–3.48)
**Current treatment**						
*TNFi*	418	62.7	171	58.8	0.253	0.84 (0.64–1.12)
*IL17i*	54	8.1	30	10.3	0.265	1.30 (0.81–2.08)
*Methotrexate (oral)*	82	12.3	35	12.0	0.908	0.97 (0.64–1.48)
*SSZ*	107	16.0	31	10.7	**0.029**	0.62 (0.40–0.95)
*Oral corticosteroid*	14	2.1	13	4.5	**0.042**	2.18 (1.01–4.70)
*Antidepressant/anxiolytic*	89	15.1	95	35.6	**< 0.001**	3.12 (2.22–4.36)

*Active at work refers to the entire sample. **Retired due to illness: the denominator includes only those patients who are not currently working

^#^ Indicates self-reported distress or a prior clinical diagnosis. Categorical variables are shown as absolute frequencies and percentages. P-values indicate the significance of the difference between sexes, calculated using the chi-square test. Odds Ratios (OR) with 95% Confidence Intervals (CI) were calculated to represent the likelihood of occurrence in females compared to males. Statistical significance was considered when *p* < 0.05

OR – Odds Ratio; 95% CI – 95% Confidence Interval; SD – Standard Deviation; IBD – Inflammatory Bowel Disease; NSAID: Nonsteroidal Anti-Inflammatory Drug; TNFi: Tumor Necrosis Factor inhibitor; SSZ: sulfasalazine IL-17i: Interleukin-17 inhibitor

**Table 2:** Sex-based comparison of clinical and functional measures in patients with AxSpA

Variable	Male Median (P25–P75)	Female Median (P25–P75)	*p*-value
Age	49 (38–57)	50 (40–57)	0.351
Age at symptoms onset	27 (19–34)	29 (21–38)	**0.001**
Age at diagnosis	35 (27–44)	37 (30–47)	**0.002**
Diagnosis delay	5 (1–13)	5 (1–13)	0.975
Disease duration	18 (10–28)	16 (9–26)	**0.041**
Enthesitis count	4 (2–9)	4 (2–11)	0.315
mSASSS	14 (5–35)	14 (4–23)	0.244
ASAS NSAID intake score	23.4 (9.5–53.6)	19.0 (7.10–50.0)	0.204
VAS PGA	5.0 (2.0–7.0)	5.0 (3.5–7.0)	**< 0.001**
VAS pain	4.0 (2.0–7.0)	5.0 (3.0–7.0)	**< 0.001**
VAS MGA	3.0 (1.0–5.0)	4.0 (2.0–6.0)	**0.006**
SJC	0.0 (0.0–1.0)	0.00 (0.0–1.0)	0.760
TJC	3.0 (2.0–6.0)	4.00 (2.0-13.5)	0.241

Data are presented as median (IQR: P25–P75). Comparisons between groups were conducted using the Mann Whitney U test for independent samples (95%CI). VAS: visual analogic scale; PGA: patient global assessment; MGA: medical global assessment; TJC: tender joint count; SJC: swollen joint count ; NSAID score: Nonsteroidal Anti-Inflammatory Drug score

In the multivariable binomial logistic regression model, male sex was significantly associated with HLA-B27 positivity (OR = 1.66; 95%CI 1.13–2.42; *p* = 0.009), smoking (OR = 2.55; 95%CI 1.34–4.88; *p* = 0.005), and hip limitation (OR = 1.85; 95%CI 1.26–2.71; *p* = 0.002). In contrast, female patients was associated with psychological distress (OR = 2.63; 95% CI: 1.85–3.85; *p* < 0.001) and with higher disease activity scores as measured by BASDAI (OR = 1.22 per unit decrease; 95% CI: 1.14–1.32; *p* < 0.001) (Fig. 2).

Table 3 presents the results of multivariable linear regression models for key outcomes in axSpA, stratified by sex. For each outcome (BASDAI, ASDAS, BASFI, BASMI, and ASQOL), the variables retained in the final models differed between men and women, highlighting distinct patterns of association. Model fit, as expressed by adjusted R², ranged from 0.398 to 0.710 across models, with similar performance in both sexes overall. However, the specific predictors and the strength of their associations varied, suggesting sex-specific pathways influencing disease outcomes in axSpA.

**Table 3:** Sex-specific multivariable linear regression models for key axial spondyloarthritis outcomes

	Male								Female						
	β		CI95% Lower		CI95% Upper		*P*-value				β		CI95% Lower	CI95% Upper	*P*-value
**BASDAI**															
*Shoulder pain*	0.556		0.207		0.904		0.002		*Shoulder pain*		0.096		0.016	1.024	0.043
									*Hip pain*		0.137		0.229	1.192	0.004
*Active peripheral*	0.370		0.008		0.733		0.045								
*BASMI*	-0.241		-0.321		-0.161		< 0.001		*BASMI*		-0.106		-0.276	-0.004	0.044
*BASFI*	0.392		0.310		0.474		< 0.001		*BASFI*		0.483		0.307	0.564	< 0.001
*ASQOL*	0.170		0.131		0.209		< 0.001								
*R* = 0.787; R²=0.619; Adjusted R² =0.608									*R* = 0.777;R²=0.604; Adjusted R²=0.595						
**ASDAS**															
*Shoulder pain*	0.235		0.032		0.438		0.023								
*Hip pain*	0.217		0.039		0.395		0.017		*Hip pain*		0.129		0.059	0.435	0.010
*Active enthesitis*	0.178		0.013		0.343		0.035								
*BASMI*	-0.081		-0.121		-0.042		< 0.001		*BASMI*		-0.130		-0.115	-0.009	0.022
*BASFI*	0.144		0.105		0.184		< 0.001		*BASFI*		0.473		0.105	0.205	< 0.001
*ASQOL*	0.060		0.041		0.078		< 0.001		*ASQOL*		0.336		0.035	0.082	< 0.001
*R* = 0.725; R²=0.525; Adjusted R² =0.511									*R* = 0.766;R²=0.587; Adjusted R²=0.574						
**BASFI**															
*Hip pain*		0.053		-0.002		0.639		0.052							
*Current working*		-0.105		-0.934		-0.294		< 0.001							
*Physically active*		-0.051		-0.599		-0.003		0.048							
*ASDAS*		0.271		0.61		1.011		< 0.001		*ASDAS*		1.007	0.659	1.320	< 0.001
*BASMI*		0.353		0.402		0.546		< 0.001		*BASMI*		0.531	0.419	0.643	< 0.001
*ASQOL*		0.399		0.181		0.257		< 0.001		*ASQOL*		0.214	0.159	0.269	< 0.001
*R* = 0.45; R²=0.713; Adjusted R² =0.710										*R* = 0.844;R²=0.712; Adjusted R²=0.708					
**BASMI**															
*Hypertension*		0.166		0.429		1.06		< 0.001							
*Smoking*		0.159		0.607		1.528		< 0.001							
*Radiographic SpA*		0.120		0.467		1.739		< 0.001							
										*Current working*		-1.233	-1.64	-0.825	< 0.001
										*Shoulder pain*		-0.954	-1.436	-0.472	< 0.001
										*Shoulder limitation*		0.712	0.095	1.33	0.024
*Hip limitation*		0.141		0.306		0.94		< 0.001							
*ASDAS*		-0.199		-0.656		-0.234		< 0.001							
*BASFI*		0.685		0.43		0.588		< 0.001		*BASFI*		0.319	0.244	0.394	< 0.001
*ASQOL*		-0.185		-0.118		-0.033		< 0.001							
*R* = 0.669; R²=0.447 Adjusted R² =0.439										*R* = 0.639; R²=0.408; Adjusted R² =0.398					
**ASQOL**															
*Psychological distress*		0.138		1.043		2.556		< 0.001							
*Current working*		-0.070		-1.439		-0.055		0.035							
*ASDAS*		0.299		1.210		2.062		< 0.001		*ASDAS*		2.288	1.394	3.182	< 0.001
*BASFI*		0.569		0.863		1.207		< 0.001		*BASFI*		0.830	0.507	1.154	< 0.001
*BASMI*		-0.128		-0.492		-0.133		< 0.001							
*R* = 0.780; R²=0.609; Adjusted R² =0.605										*R* = 0.815. R² = 0.664. and adjusted R² = 0.619					

Results of multivariable linear regression analyses for five outcome variables (BASDAI, ASDAS, BASFI, BASMI, and ASQOL) in male and female patients with axial spondyloarthritis. For each predictor retained in the final model, the standardized regression coefficient (β), 95% confidence interval (CI), and p-value are shown. Only statistically significant predictors (*p* < 0.05) are included. Coefficients reflect the unique contribution of each variable to the outcome after adjustment for other predictors in the model. Model fit is expressed using R, R², and adjusted R²

## Discussion

This study provides significant evidence of sex-specific differences in the clinical presentation, disease burden, and predictors of outcomes in axSpA using a large, nationwide Brazilian cohort. Our findings reinforce the concept that axSpA is not a homogeneous disease across sexes and suggest that personalized approaches to diagnosis and management are warranted.

The growing interest in investigating the presentation and evolution of axSpA in women has allowed us to find anatomical, clinical and functional differences that help to better characterize the disease. Anatomical factors, such as the shape of the pelvis and the different mechanical overloads observed in women, contribute to highlighting these differences [16, 17];

Sex-related differences in axSpA generally characterize distinct clinical presentations. While men frequently present more classic axial presentation associated with HLA-B27 positivity, women tend to have a higher frequency of peripheral involvement, upper limb arthritis, and dactylitis, and more often receive diagnoses of psoriatic arthritis or peripheral SpA, suggesting greater clinical heterogeneity among female patients with axSpA [18]. Our study also reinforced that lifestyle-related variables, especially alcoholism and smoking in men, played a role in disease evolution, as was previously shown in an international study [19].

Another significant difference between the sexes in axSpA is related to the disease assessment instruments. Women in our cohort reported significantly higher disease activity scores, as well as greater functional impairment and poorer quality of life compared to men, despite men having greater impairment in spinal mobility. This reinforces the perception of a more burdensome disease experience in female patients, especially in patient-reported measures such as fatigue and pain. While the BASDAI is entirely subjective, ASDAS incorporates C-reactive protein (CRP) as an objective marker, which has been thought to mitigate sex-related differences [20]. However, in our cohort, CRP levels were similar between men and women, yet ASDAS-CRP scores remained higher in women. A recent systematic review highlighted consistent evidence that women with axSpA report higher disease activity than men, particularly in subjective domains such as fatigue, pain, and functional limitations. Although the data on the mechanisms behind these differences are limited, they may stem from a combination of socio-cultural coping factors and biological influence, such as variations in hormonal profiles and immune responses, that contribute to sex dimorphism [21].

Importantly, the greater functional limitation and impaired quality of life reported by women draw attention to the broader impact of the disease on daily functioning and well-being, probably reflecting a complex interplay of biological and psychosocial factors. Supporting this notion, it was observed that spinal structural damage had limited explanatory power over physical function compared to disease activity and mobility, suggesting that subjective burden may indeed outweigh radiographic findings in shaping the patient experience, particularly in women [22]. This combined analysis of BASFI and ASQoL scores highlights a relevant sex-related paradox: although female patients reported significantly worse functional status and poorer quality of life, these impairments occurred despite better spinal mobility. This dissociation suggests that factors beyond axial structural damage, such as pain perception, peripheral manifestations, and psychological burden, may disproportionately affect functional capacity and overall well-being in women with axSpA. This contrasts with results from a Swedish study [23], which found no significant difference in BASFI scores between sexes in a cohort of 353 patients with r-axSpA. This may reflect differences in disease subtype and the higher burden of psychological distress among women, which can influence BASFI scores.

The associations observed in the regression models for the disease assessment instruments showed very interesting results when these models were stratified by sex. Hypertension and smoking were independently associated with worse spinal mobility (BASMI), and physical activity was negatively associated with functional limitation (BASFI) in men. In this group, ASQoL was shaped not only by functional and mobility indices but also by psychological distress, which was not observed in female patients. This may be explained by limited contrast in the model or possibly because its impact was indirectly captured through other variables such as BASFI and ASDAS. In contrast, women showed a more compact set of predictors concentrated on pain and function. All five outcomes were primarily influenced by core musculoskeletal variables such as BASFI, BASMI, ASDAS, and joint pain. No comorbidities or behavioral factors appeared as independent predictors in women. These findings are supported by the COMOSPA study, where cardiometabolic comorbidities were more frequent and more strongly associated with impaired physical function and structural outcomes in men, while psychosocial factors were more prevalent and impactful in women [24].

Although overall activity levels were similar, more men exercised 4–7 times per week. This was independently associated with lower disease activity, particularly in men. While the cross-sectional design limits causal inference, the finding reinforces the therapeutic role of regular exercise in axSpA management [25].

Treatment profiles were largely similar between sexes. However, a higher proportion of women had previously used TNF inhibitors and were currently on IL-17 inhibitors, suggesting a trend toward greater therapeutic escalation in female patients. These patterns may reflect more difficult disease control in women. Possible factors include subjective burden, comorbidities, or distinct axSpA phenotypes that influence treatment choices. Recent studies have also found lower response rates to bDMARDs [26] and shorter survival to bDMARDs [27, 28] in patients with axSpA.

This study has several limitations. One key limitation is how we defined psychological distress. We chose a broader definition that included self-report, previous medical diagnosis, or current use of antidepressants or anxiolytics. We did this because we wanted to capture a wide range of patients who might be experiencing psychological difficulties, even if they hadn’t received a formal diagnosis. While this approach reflects the variability we see in clinical practice, it also means that we group together people with different types and levels of distress. This could have affected how strongly psychological distress was linked to other outcomes. That said, we believe this inclusive approach provides a more realistic picture of what’s happening in routine care, especially given differences in mental health screening across centers. We recognize that having more detailed psychiatric assessments would have allowed for a more refined analysis, and we encourage future research in this direction.

Another limitation of our study is the absence of data on fibromyalgia frequency. This variable was not chosen because peripheral involvement, including enthesitis, is quite common in our patients with axSpA, and there is still considerable overlap between the endpoints of fibromyalgia and enthesitis, making it difficult to accurately distinguish between the two clinical situations. A recent study that evaluated 526 patients with axSpA, of which 38% were diagnosed with fibromyalgia, corroborates this statement [29]. The absence of formal fibromyalgia diagnosis in our dataset is an important limitation, given its known influence on patient-reported outcomes in axial spondyloarthritis. Fibromyalgia, particularly through mechanisms of central sensitization, can substantially amplify symptoms such as pain, fatigue, and functional impairment, independently of objective inflammatory activity. This overlap could lead to misclassification of disease burden, especially among patients with high PROMs but low inflammation markers. While the presence of widespread enthesitis may partially reflect underlying central sensitization, it is not a substitute for a clinical diagnosis of fibromyalgia. The inability to adjust for fibromyalgia may have biased the observed associations, particularly in women, where fibromyalgia is more prevalent and may contribute disproportionately to higher BASDAI, ASQoL, and psychological distress scores.

Because all participants were recruited from 17 tertiary referral centers, the cohort likely includes a disproportionate number of patients with more severe, refractory or complex presentations. This referral pattern may limit the external validity of our findings, particularly in relation to patients managed in primary care or community settings, where disease severity, comorbidity profiles, and access to specialized care may differ substantially.

Finally, it is important to note that the present findings are based on a binary classification of sex and do not include individuals from LGBTQIA + or transgender populations. Currently, data regarding the clinical presentation, disease burden, psychosocial impact, and treatment response in these groups are scarce. Further research is urgently needed to explore how gender diversity may influence the experience and management of axSpA, including potential differences in healthcare access, diagnostic delay, and psychosocial outcomes.

Nevertheless, our registry has several strenghts, including the use of validated outcome measures and standardized data collection procedures across centers, which reinforce the reliability of our findings. Furthermore, the large sample size and the multicenter, geographically diverse cohort reinforce the external validity of our results within a real-world Brazilian context.

In conclusion, our findings demonstrated that axSpA may be expressed through distinct biological and psychosocial pathways in men and women. While men were more likely to express markers of structural disease, such as HLA-B27 positivity and hip involvement, and showed a disease burden shaped by comorbidities and lifestyle factors, such as smoking, women exhibited a profile dominated by pain, functional impairment, and psychological distress. These patterns were consistent across descriptive and multivariable analyses, underscoring a fundamental divergence in how axSpA manifests and impacts patients’ lives. Rather than viewing sex as a confounding factor, our results suggest it acts as a lens through which illness is experienced. Integrating this perspective into care strategies is essential for delivering truly personalized and equitable treatment. In line with our earlier nationwide study, which reported more peripheral involvement and higher patient-reported activity in women and greater axial damage in men [30], the present analysis focusing on axSpA confirms these sex-specific patterns and adds new insights by identifying psychosocial and comorbidity-related pathways underlying these differences. Future research should explore longitudinal trajectories of disease activity and psychosocial burden, incorporating fibromyalgia screening tools and objective measures of inflammation such as imaging and biomarkers. Registry-based studies could also investigate the impact of treatment strategies on long-term quality of life and work productivity, particularly among underrepresented subgroups.

## Supplementary Information

Below is the link to the electronic supplementary material.

Supplementary Material 1

## Data Availability

The datasets used and/or analysed during the current study are available from the corresponding author on reasonable request.
