# Supplementary material for: Impact of sex in axial spondyloarthritis: insights from the Brazilian Registry of Spondyloarthritis
Source: Adv Rheumatol. 2026 Feb 12;66:25. doi: 10.1186/s42358-026-00525-3 (PMC13488764; doi:10.1186/s42358-026-00525-3)
Supplement: Supplementary file 1 [file 42358_2026_525_MOESM1_ESM.docx]

Supplementary table: Variables associated with disease outcomes in patients with axial spondyloarthritis, stratified by sex

|  | **Male** | | **Female** | |
| --- | --- | --- | --- | --- |
|  | **Mean difference (95% CI)** | **p-value** | **Mean difference (95% CI)** | **p-value** |
| **BASDAI** | | | | |
| Psychological distress | 0.70 | **0.006** | 0.30 | 0.324 |
| Not currently working | 0.40 | **0.034** | 0.20 | 0.632 |
| Physical inactivity) | 0.70 | **<0.001** | 1.10 | **0.001** |
| Shoulder pain | 2.20 | **<0.001** | 1.80 | **<0.001** |
| Shoulder limitation | 1.60 | **<0.001** | 2.10 | **<0.001** |
| Hip pain | 1.70 | **<0.001** | 2.00 | **<0.001** |
| Hip limitation | 1.00 | **<0.001** | 1.50 | **<0.001** |
| Active peripheral | 1.80 | **<0.001** | 0.80 | **0.044** |
| Active dactylitis | 1.10 | 0.075 | 3.50 | **0.026** |
| TNFi failure | 0.40 | **0.035** | 0.40 | 0.180 |
| **ASDAS-CRP** |  |  |  |  |
| Radiographic SpA | 0.10 | 0.446 | 0.50 | **0.037** |
| Physical inactivity | 0.30 | **<0.001** | 0.40 | **0.001** |
| Shoulder pain | 0.70 | **<0.001** | 0.70 | **<0.001** |
| Shoulder limitation | 0.50 | **<0.001** | 0.70 | **<0.001** |
| Hip pain | 0.70 | **<0.001** | 0.70 | **<0.001** |
| Hip limitation | 0.30 | **<0.001** | 0.60 | **<0.001** |
| Active peripheral | 0.60 | **<0.001** | 0.20 | 0.174 |
| Active dactylitis | 1.10 | 0.075 | 3.50 | **0.026** |
| Active enthesitis | 0.49 | **<0.001** | 0.00 | 0.781 |
| TNFi failure | 0.20 | **0.046** | 0.10 | 0.278 |
| **BASMI** |  |  |  |  |
| HLA-B27 | 0.50 | **0.020** | 0.30 | 0.307 |
| Radiographic SpA | 1.90 | **<0.001** | 0.50 | 0.188 |
| Psoriasis | 2.30 | **0.004** | 0.05 | 0.943 |
| Diabetes | 1.10 | **<0.001** | 0.60 | 0.152 |
| Hypertension | 1.40 | **<0.001** | 1.00 | **<0.001** |
| Obesity | 0.69 | **0.034** | 0.40 | 0.350 |
| Smoking | 1.40 | **<0.001** | 0.30 | 0.612 |
| Not currently working | 1.60 | **<0.001** | 1.70 | **<0.001** |
| Shoulder pain | 0.40 | **0.034** | 0.10 | 0.553 |
| Shoulder limitation | 1.10 | **<0.001** | 0.70 | **0.030** |
| Hip pain | 0.60 | **<0.001** | 0.40 | 0.121 |
| Hip limitation | 1.20 | **<0.001** | 1.30 | **<0.001** |
| TNFi failure | 0.60 | **0.003** | 0.40 | 0.074 |
| **BASFI** |  |  |  |  |
| HLA-B27 | 0.60 | **0.049** | 0.10 | 0.740 |
| Radiographic SpA | 1.00 | **0.045** | 0.10 | 0.800 |
| Diabetes | 1.00 | **0.003** | 1.60 | **0.003** |
| Hypertension | 1.00 | **<0.001** | 1.10 | **0.005** |
| Psychological distress | 0.30 | 0.264 | 0.80 | **0.037** |
| Obesity | 0.90 | **0.023** | 0.90 | 0.198 |
| Smoking | 1.00 | **0.009** | 1.60 | **0.030** |
| Not currently working | 2.10 | **<0.001** | 1.80 | **<0.001** |
| Physical inactivity | 1.10 | **<0.001** | 1.00 | **0.005** |
| Shoulder pain | 2.10 | **<0.001** | 1.50 | **<0.001** |
| Shoulder limitation | 2.30 | **<0.001** | 1.90 | **<0.001** |
| Hip pain | 2.00 | **<0.001** | 1.90 | **<0.001** |
| Hip limitation | 1.90 | **<0.001** | 2.30 | **<0.001** |
| Active peripheral | 1.60 | **<0.001** | 0.40 | 0.198 |
| Active enthesitis | 0.70 | **0.014** | 0.70 | 0.105 |
| TNFi failure | 0.90 | **<0.001** | 0.00 | 0.905 |
| **ASQOL** |  |  |  |  |
| HLA-B27 | 1.00 | **0.021** | 0.00 | 0.907 |
| Psoriais | 5.00 | **0.028** | 4.00 | **0.031** |
| Psychological  distress | 2.00 | **0.001** | 2.00 | **0.009** |
| Hypertension | 1.00 | **0.025** | 0.00 | 0.652 |
| Obesity | 2.00 | **0.027** | 3.00 | **0.009** |
| Smoking | 2.00 | **0.011** | 2.00 | 0.219 |
| Not currently working | 3.00 | **<0.001** | 2.00 | **<0.001** |
| Physical inactivity) | 2.00 | **<0.001** | 3.00 | **<0.001** |
| Shoulder pain | 5.00 | **<0.001** | 3.00 | **<0.001** |
| Shoulder limitation | 4.00 | **<0.001** | 3.00 | **<0.001** |
| Hip pain | 3.00 | **<0.001** | 4.00 | **<0.001** |
| Hip limitation | 3.00 | **<0.001** | 4.00 | **<0.001** |
| Active peripheral | 3.00 | **<0.001** | 1.00 | 0.085 |
| Active dactylitis | 4.00 | **0.027** | 6.00 | **0.033** |
| Active enthesitis | 2.00 | **0.003** | 1.00 | 0.635 |
| TNFi failure | 2.00 | **<0.001** | 1.00 | 0.174 |

*Mann-Whitney U test, 95%CI. BASDAI: Bath Ankylosing Spondylitis Disease Activity Index; ASDAS-CRP: Ankylosing Spondylitis Disease Activity Score with C-reactive protein; BASFI: Bath Ankylosing Spondylitis Functional Index; BASMI: Bath Ankylosing Spondylitis Metrology Index; ASQoL: Ankylosing Spondylitis Quality of Life; HLA-B27: Human leukocyte antigen B27 ; TNFi : Tumor Necrosis Factor inhibitor*
